# Largest Magnetic Moments in the Half-Heusler Alloys XCrZ (X = Li, K, Rb, Cs; Z = S, Se, Te): A First-Principles Study

**DOI:** 10.3390/ma10091078

**Published:** 2017-09-14

**Authors:** Xiaotian Wang, Zhenxiang Cheng, Guodong Liu

**Affiliations:** 1 School of Physical Science and Technology, Southwest University, Chongqing 400715, China; wangxt45@126.com or xiaotianwang@swu.edu.cn; 2 College of Physics and Information Technology, Chongqing Normal University, Chongqing 401331, China; 3 Institute for Superconducting & Electronic Materials (ISEM), University of Wollongong, Wollongong 2500, Australia; cheng@uow.edu.au

**Keywords:** half-Heusler alloys, intermetallic materials, first-principles calculation

## Abstract

A recent theoretical work indicates that intermetallic materials LiMnZ (Z = N, P) with a half-Heusler structure exhibit half-metallic (HM) behaviors at their strained lattice constants, and the magnetic moments of these alloys are expected to reach as high as 5 μ_B_ per formula unit. (Damewood et al. *Phys. Rev. B* **2015**, *91*, 064409). This work inspired us to find new Heusler-based half-metals with the largest magnetic moment. With the help of the first-principles calculation, we reveal that XCrZ (X = K, Rb, Cs; Z = S, Se, Te) alloys show a robust, half-metallic nature with a large magnetic moment of 5 μ_B_ at their equilibrium and strained lattice constants in their most stable phases, while the excellent HM nature of LiCrZ (Z = S, Se, Te) alloys can be observed in one of their metastable phases. Moreover, the effects of uniform strain in LiCrZ (Z = S, Se, Te) alloys in type II arrangement have also been discussed.

## 1. Introduction

In recent years, the fast development of spintronics [1] has caused more and more concern for researchers. Extensive applications (e.g., single spin electron sources [2] and spin injections [3]) have been envisaged [4]. One ideal choice for spintronics is the source of the spin-polarized charge carriers (SPCC). In this quest for materials, half-Heusler half-metallic alloys [5] are a noticeable family of intermetallic materials with 1:1:1 composition and have fully SPCC at the Fermi level.

The half-Heusler family has become one of the research hot-spots in intermetallic materials systems because the concept of half-metallic (HM) behaviors arose from the theoretical calculations by de Groot et al. for the well-known NiMnSb [6] in the 1980s. Then, quite a lot of half-Heusler alloys [7,8,9,10,11,12,13,14,15] had been predicted to be HM materials (HMMs). Recently, the electronic, magnetic, and stability properties were systematically investigated by first principles calculation in half-Heusler alloys of LiMnZ (Z = N, P, Si) [16]. Damewood et al. found that these LiMnZ (Z = N, P, Si) alloys show HM behaviors with large semiconducting-type band-gaps and magnetic moments (>3 μ_B_ per formula unit) at their strained lattice constants. To our best knowledge, based on the the Slater-Pauling (S-P) and generalized electron-filling rules [17,18,19,20], the largest magnetic moment of the half-Heusler-type alloy should be 5 μ_B_ per formula unit. Due to the large semiconducting-type band-gaps and the large magnetic moments, LiMnZ (Z = N, P, Si) alloys may be good candidates for spintronic materials for devices operating at or above room temperature.

Based on the above information, it is necessary for us to further explore new HM half-Heuselr alloys, XCrZ (X = Li, K, Rb, Cs; Z = S, Se, Te), with the largest magnetic moment (5 μ_B_ per formula unit) and large semiconducting-type band-gaps. In this work, first-principles calculations have been used to exhibit a theoretical study of the structural, electronic, magnetic, and HM properties of the XCrZ (X = Li, K, Rb, Cs; Z = S, Se, Te) half-Heusler alloys in three possible arrangements. 

## 2. Computational Details

The electronic-structure and magnetism calculations were performed via CASTEP code on the basis of the pseudo-potential method with a plane-wave basis set [21,22]. The ultrasoft pseudo-potential [23] has been used to describe the interactions between the atomic core and the valence electrons. A most common generalized gradient approximation (GGA) [24] has been selected to describe the electron exchange-correlation. A plane-wave basis set cut-off of 450 eV and a mesh of 12 × 12 × 12 k-points was used for Brillouin zone integrations. The convergence tolerance for the calculations was selected as a difference in total energy within 1 × 10^−6^ eV·atom^−1^.

To determine the real-space bonding analysis, the electron localization function (ELF) was calculated using the CASTEP code. The ELF is a real-space indicator of the extent to which electrons are localized and display a strong Pauli repulsion, and therefore it can locate bonding and non-bonding electron pairs in the real-space of the crystal-structure [25,26].

## 3. Results and Discussion

**Figure 1:**
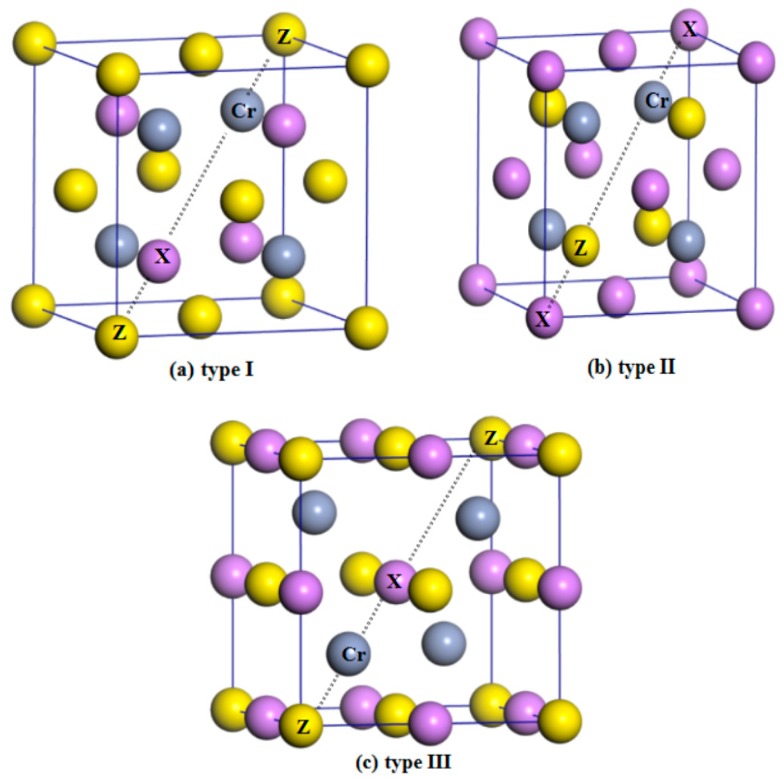
Crystal structures of the three arrangements within the half-Heusler alloys XCrZ (X = Li, K, Rb, Cs; Z = S, Se, Te) noted as type I (**a**); type II (**b**) and type III (**c**).

**Figure 2:**
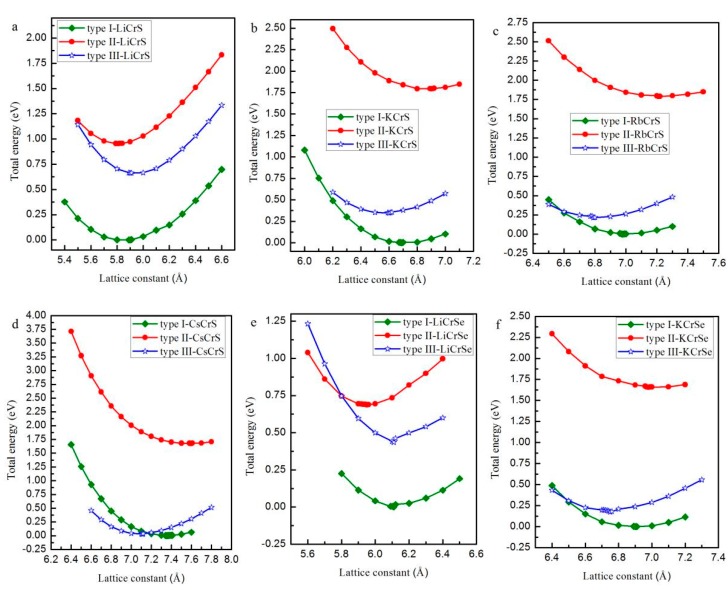

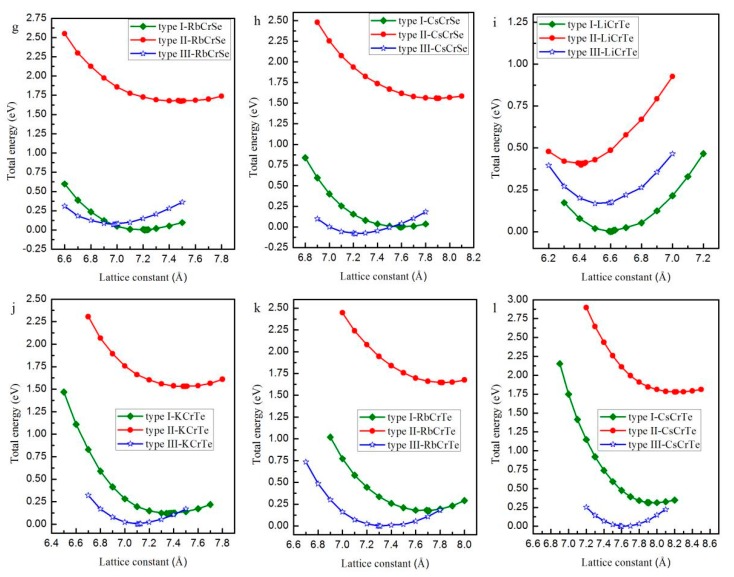
The total energy as a function of the lattice constant in the three atomic arrangements type I, type II, and type III for half-Heusler alloys LiCrS (**a**); KCrS (**b**); RbCrS (**c**); CsCrS (**d**); LiCrSe (**e**); KCrSe (**f**); RbCrSe (**g**); CsCrSe (**h**); LiCrTe (**i**); KCrTe (**j**); RbCrTe (**k**) and CsCrTe (**l**), respectively.

Normally, half-Heusler alloys have a formula of XYZ. In this work, X is the Li, K, Rb, and Cs atoms, Y is the Cr atom, and Z is the main-group-element atoms S, Se, and Te. As shown in Figure 1, in the half-Heusler alloys XCrZ with the C1_b_ structure, three possible arrangements have been taken into consideration: type I = [4*c* (0.25, 0.25, 0.25), 4*d* (0.75, 0.75, 0.75), 4*a* (0, 0, 0)], type II = [4*a* (0, 0, 0), 4*d* (0.75, 0.75, 0.75), 4*c* (0.25, 0.25, 0.25)] and type III = [4*b* (0.5, 0.5, 0.5), 4*d* (0.75, 0.75, 0.75), 4*a* (0, 0, 0)]. To obtain the equilibrium lattice structures of the XCrZ, the geometry optimization is firstly performed in their three possible arrangements. The achieved total energy-lattice constant curves for XCrZ are shown in Figure 2. Obviously, KCrS, RbCrS, CsCrS, KcrSe, and RbCrSe alloys have their lowest energies in type I instead of type II and III. For the CsCrSe, KCrTe, RbCrTe, and CsCrTe (LiCrS, LiCrSe, and LiCrTe) alloys, the structure of type III (type II), with the lowest energy, is the most stable among the three arrangements.

Table 1 shows for all XCrZ (X = Li, K, Rb, Cs; Z = S, Se, Te) the calculated total magnetic moments and the sizes of the semiconducting-type band-gaps. For the semiconducting-type band-gaps, the Fermi level (*E_F_*) just falls within the gap in the spin-down band, indicating semiconductor properties. As can be observed in Table 1, the values of the total magnetic moment per formula unit (M_t_) are 5 μ_B_ for XCrZ (X = K, Rb, Cs; Z = S, Se, Te) in their most stable phases (arrangements). However, the calculated magnetic moments per formula unit for LiCrZ (Z = S, Se, Te) are not the integral Bohr magneton for the most stable phase. As is known, for the Heusler-type HMMs, their calculated M_t_ is usually an integer value [27,28,29,30]. The non-inter values of M_t_ for LiCrZ (Z = S, Se, Te) in type II indicate that they are not HMMs in the most stable phase. Also, as shown in Table 1, all XCrZ alloys have very large semiconducting-type band-gaps (>2 eV) in their most stable phase, except for LiCrZ (Z = S, Se, Te). It means that they may maintain their magnetic and HM behaviors at room temperature.

**Figure 3:**
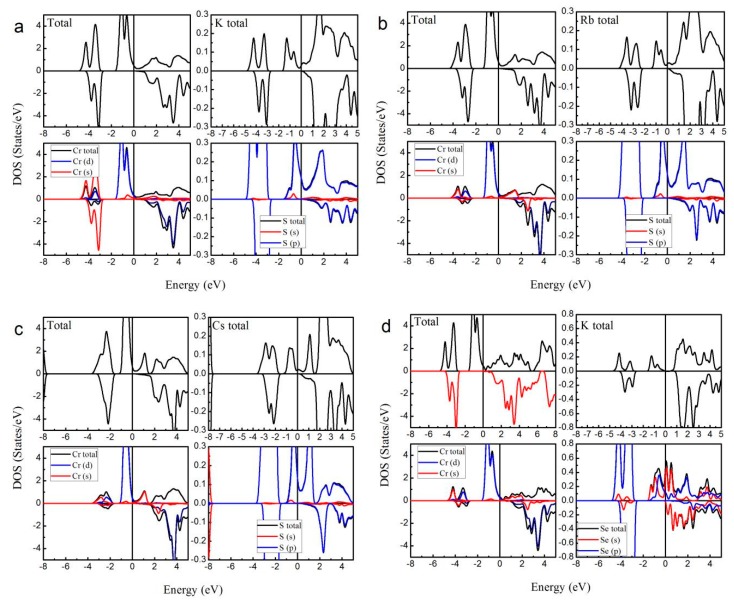

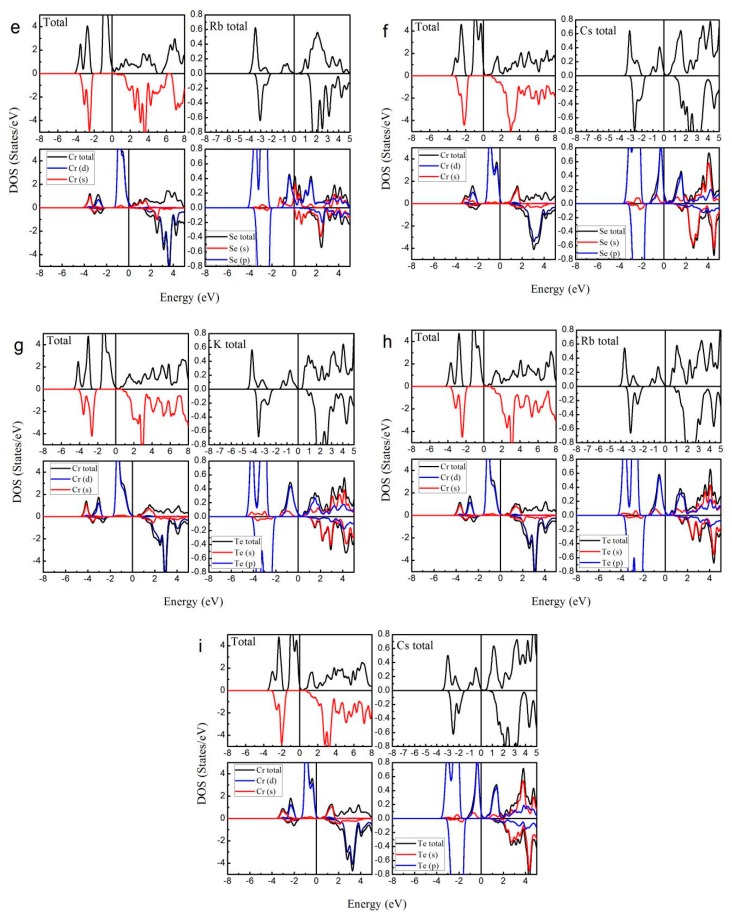
Calculated total and atom-projected density of states (DOS) for (**a**) type I KCrS; (**b**) type I RbCrS; (**c**) type I CsCrS; (**d**) type I KCrSe; (**e**) type I RbCrSe; (**f**) type III CsCrSe; (**g**) type III KCrTe; (**h**) type III RbCrTe; and (**i**) type III CsCrTe. (The Fermi level *E_F_* was set as X = 0).

To further confirm the possible half-metallicity of XCrZ (X = K, Rb, Cs; Z = S, Se, Te), we show in Figure 3 the calculated total and atom-projected DOS for KCrS, RbCrS, CsCrS, KCrSe, and RbCrSe in the type I arrangement and CsCrSe, KCrTe, RbCrTe, and CsCrTe in the type III arrangement, respectively. Obviously, it can be found that all these mentioned alloys show half-metallic behaviors: in the majority spin (spin-up) channel, the energy bands exhibit a metallic overlap with the *E_F_*, whereas in the minority spin (spin-down) direction, an energy gap is opened and the *E_F_* locates within the gap.

It is common sense that the DOS can be widely used to discuss the bonding/anti-bonding states and the gap formation, and we should also point out that a similar analytical approach used in this work can be observed in [31,32].

Figure 3 shows the total density of states (TDOS) and the partial density of states (PDOS) of XCrZ (X = K, Rb, Cs; Z = S, Se, Te) in the most stable phase. Obviously, in both spin channels, the main contributions to the TDOS around the Fermi level arose from the 3d states of the Cr atom. The X and Z atoms have a rather lower PDOS than the Cr atom. 

As shown in Figure 3, in the spin-up channel the main peaks of the Cr and Z atoms occurred in the range from −2 eV to 0 eV, and −4 eV to −2 eV, respectively. Meanwhile, between the −4 eV and −2 eV states, similar-shaped, hybridized peaks can also be found in the Cr atoms. In the spin-down channel, in the same energy range (−4 eV~−2 eV), for the Cr and Z atoms, such hybridized peaks appeared at the same time. Therefore, the hybridization between the Cr and Z atoms that formed strong bonding states range from −4 eV to −2 eV. Above the *E_F_*, in the spin-down channel, the anti-bonding can be found at around 3 eV, and in the spin-up channel, no opposite energy states are observed. Moreover, similar to the LiMnZ alloys [16], the bonding-antibonding states led to the formation of a semiconducting-type band-gap in the spin-down channel. 

For the type I and type III arrangements, the semiconducting-type band-gaps of the XCrZ (X = K, Rb, Cs; Z = S, Se, Te) alloys are very large. However, compared to the type I and type III arrangements, the type II arrangement does not form a large semiconducting-type band-gap (see Table 1) because the Cr and Z are second neighbors in a cubic environment.

In addition, as mentioned above, the calculated M_t_, 5 μ_B_ for XCrZ (X = K, Rb, Cs; Z = S, Se, Te) follows the modified S-P rule recently presented by Damewood et al. [16],

M_t_ = (Z_t_ − 8)·μ_B_, (1)

here Z_t_ is the number of total of valence electrons in XCrZ (X = K, Rb, Cs; Z = S, Se, Te), respectively.

**Figure 4:**
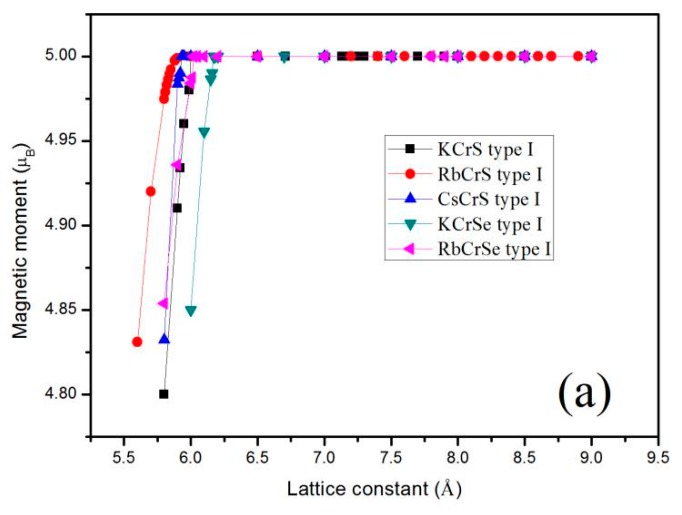

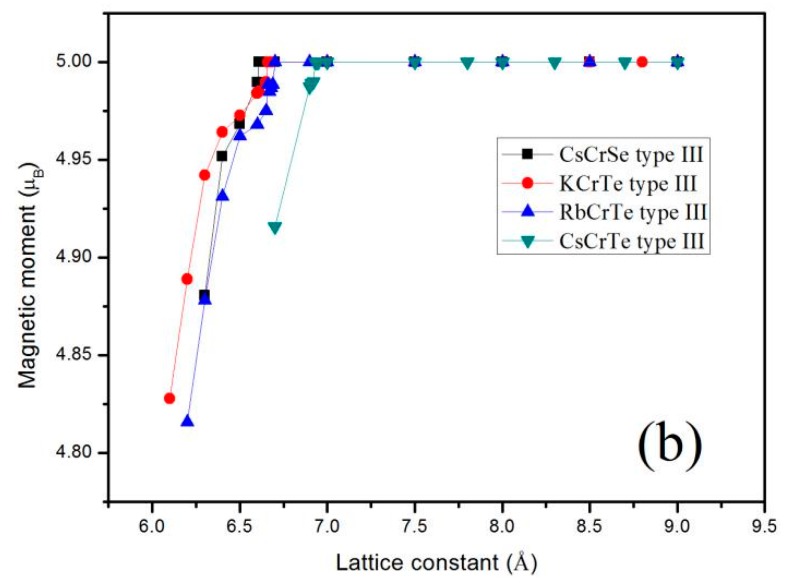
Calculated total magnetic moments as functions of the lattice constant for KCrS, RbCrS, CsCrS, KCrSe, RbCrSe (**a**) and CsCrSe, KCrTe, RbCrTe, CsCrTe (**b**), respectively, with their most stable phase.

Furthermore, the total M_t_ for the XCrZ (X = K, Rb, Cs; Z = S, Se, Te) alloys at their strained lattice constants with the most stable phase has been calculated, and the results are shown in Figure 4. Obviously, for all these mentioned alloys, the total M_t_ of 5.00 μ_B_/f.u remained constant within an expansion and contraction of less than 0.01 µ_B_ over a large range of lattice constant values. That is to say, the half-metallic behavior of these alloys is quite robust. When the lattice constants are compressed to the critical value 5.99 Å for KCrS, 5.84 Å for RbCrS, 5.92 Å for CsCrS, 6.16 Å for KCrSe, 6.01 Å for RbCrSe, 6.60 Å for CsCrSe, 6.65 Å for KCrTe, 6.69 Å for RbCrTe, and 6.92 Å for CsCrTe, respectively, the semiconducting-type band-gap in the spin-down channel closed and thus the integer value of the total magnetic moments disappeared.

Previous studies have shown that ternary alloys, including the Li atom, are good candidates in optoelectronic and spintronic applications [33,34]. Although the LiCrZ alloys presented in the current work are not HMMs (see Table 1) in the most stable arrangement (type I), the other arrangements (type II and III) should also be reported here to check the electronic, magnetic, and half-metallic properties. We hope to search for a metastable (type II or III) of the LiCrZ (Z = S, Se, Te) alloys exhibiting HM behavior with the largest magnetic moment (5 μ_B_) and a large semiconducting-type band-gap (>2 eV).

**Figure 5:**
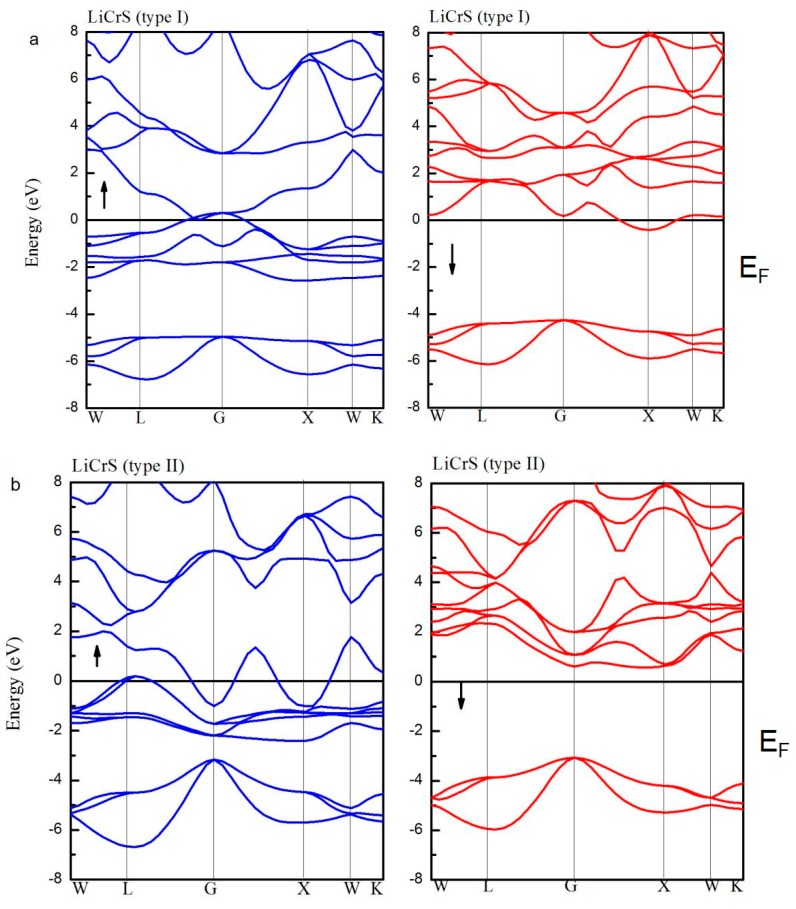

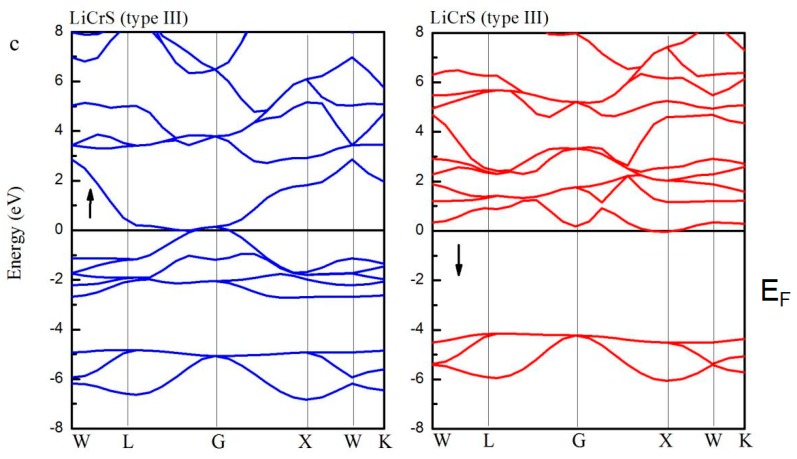
The spin-up (blue lines) and spin-down (red lines) band structures for LiCrS alloy in the three atomic (type I (**a**); type II (**b**); and type III (**c**)) arrangements. (The Fermi level *E_F_* was set as Y = 0).

Figure 5 shows the spin-up (blue lines) and spin-down (red lines) band structures for the LiCrS alloy in the three atomic (type I, type II, and type III) arrangements. Definitely, in the type I and III arrangements, in both the spin channels, the *E_F_* overlaps with the energy bands. However, the LiCrS in the type II arrangement exhibits a half-metallic nature, namely, the majority spin electrons show metallic behaviors and the minority spin electrons exhibit semiconducting properties. Similar behaviors were also discovered in the LiCrSe and LiCrTe alloys. The indirect semiconducting-type band-gaps of the LiCrZ (Z = S, Se, Te) alloys in the type II arrangement are 3.62 eV, 3.15 eV, and 2.10 eV, respectively, and are also listed in Table 1. 

**Figure 6:**
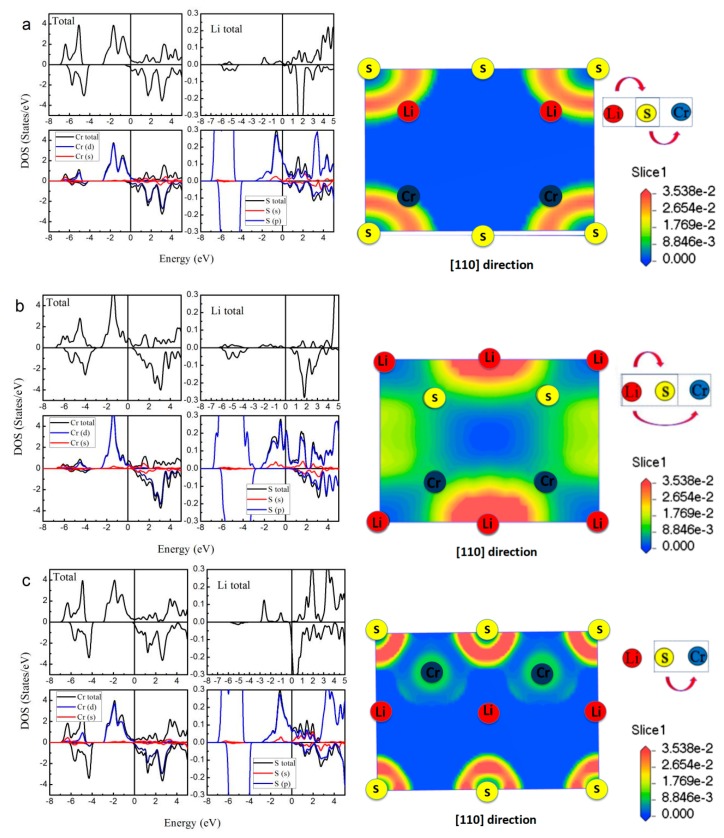
Calculated total and atom-projected DOS, and the electron localization function (ELF) graphs of the LiCrS alloy in the three atomic arrangements: type I (**a**); type II (**b**); and type III (**c**). (The Fermi level *E_F_* was set as X = 0).

As a representative of all the LiCrZ alloys, in Figure 6, we display the calculated total and atom-projected DOS, and the ELF graphs of the LiCrS alloy in the three atomic arrangements (type I, type II, and type III). For the type II arrangement, in the minority spin channel, the antibonding peak is shifted high above *E_F_* due to the exchange splitting [18], whereas for the case of the type I and III arrangements, the minority DOS, together with the energy gap, moves to low energy. Moreover, we also perform the ELF maps project on the (1 1 0) plane of the LiCrS in types I, II, and III, respectively. The high- and low- ELF values in the graphs of ELF correspond to areas of localized electrons and the area around the maxima, respectively [35]. As shown in Figure 6a,c, for the type I and type III arrangements, the regions of the highest ELF value are all around the main-group S atom along the S-Cr bound axes, indicating their sharing behavior and the occurrence of the covalent bond. We should note that, for the type I and III arrangements, the S and Cr atoms are nearest neighbors and show strong S-Cr covalent-hybridization. However, for the type II arrangement, the S and Cr atoms sit by the second neighbor sites and show nearly no S-Cr covalent-hybridization. 

In the half-Heusler alloys LiCrZ (Z = S, Se, Te), through the DOS and the ELF maps, we can summarize the electronic structure into the following two features: (i) under the strong S-Cr covalent-hybridization, LiCrZ (Z = S, Se, Te) alloys with both type I and type III arrangements show typically metallic band structure; (ii) under less S-Cr covalent-hybridization, LiCrZ with type II arrangement takes on excellent half-metallic band behavior with largest magnetic moment 5 μ_B_ at the equilibrium lattice constant.

**Figure 7:**
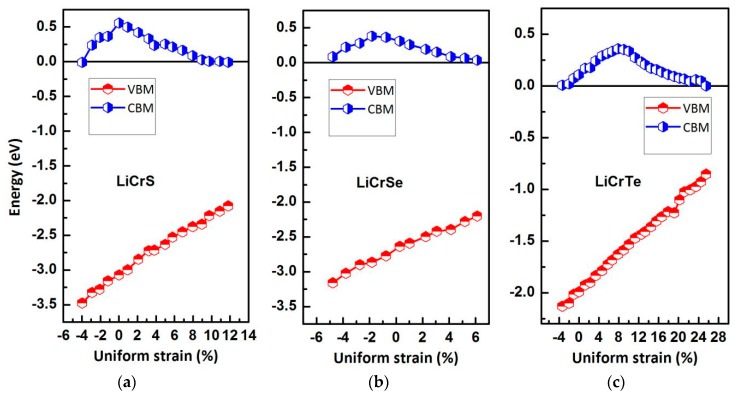
Calculated valence band maximum (VBM) and conduction band minimum (CBM) as a function of the lattice constants for the LiCrS (**a**), LiCrSe (**b**) and LiCrTe (**c**) alloys in the type II arrangement. The black circle and the red circle represent the VBM and CBM, respectively. (The Fermi level *E_F_* was set as Y = 0).

In the spintronic devices, the Heusler type HM multilayers or thin films are often touched by researchers; however, the actual and ideal lattice constants are usually inconsistent. The change of the lattice constants will lead to significant changes of the electronic, magnetic, and HM properties of the equilibrium state. Hence, we need to examine the HM stability for the LiCrZ (Z = S, Se, Te) in the type II arrangement at the strained lattice constants. The band-structure calculations at the strained lattice constants were performed for the LiCrZ (Z = S, Se, Te) alloys in the type II arrangement. In this discussion, the values of CBM and VBM for the LiCrZ (Z = S, Se, Te) alloys in the minority spin channel have been recorded to show the HM behavior for clarity, as plotted in Figure 7. Obviously, the HM states are kept for the lattice constants of 5.57–6.50 Å for LiCrS, 5.71–6.36 Å for LiCrSe, and 6.15–7.94 Å for LiCrTe, respectively. That is, these three alloys can maintain their half-metallicity when their lattice constants are changed by −4.2% to 11.68%, −4.83% to 6%, and −3.45% to 24.64% relative to the equilibrium lattice constants.

**Figure 8:**
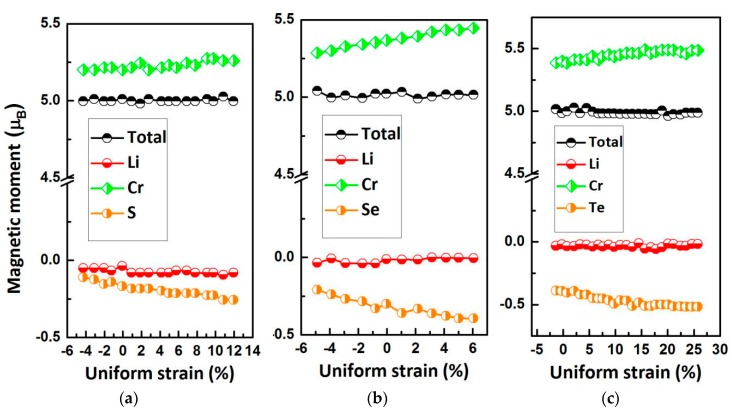
Total and atomic magnetic moments as functions of the lattice constants for the LiCrS (**a**), LiCrSe (**b**) and LiCrTe (**c**) alloys in type II arrangement.

In Figure 8, we show the relationship between the M_t_ and the lattice constant of type II LiCrZ (Z = S, Se, Te). Obviously, the total M_t_ is always a fixed integer value 5 μ_B_ in the whole variational range. The atomic M_t_ of Cr and Z are sensitive to lattice distortion. The M_t_ of the Cr atom increases with increasing lattice constants, whereas for the Z (Z = S, Se, Te) atom they continuously decrease.

**Figure 9:**
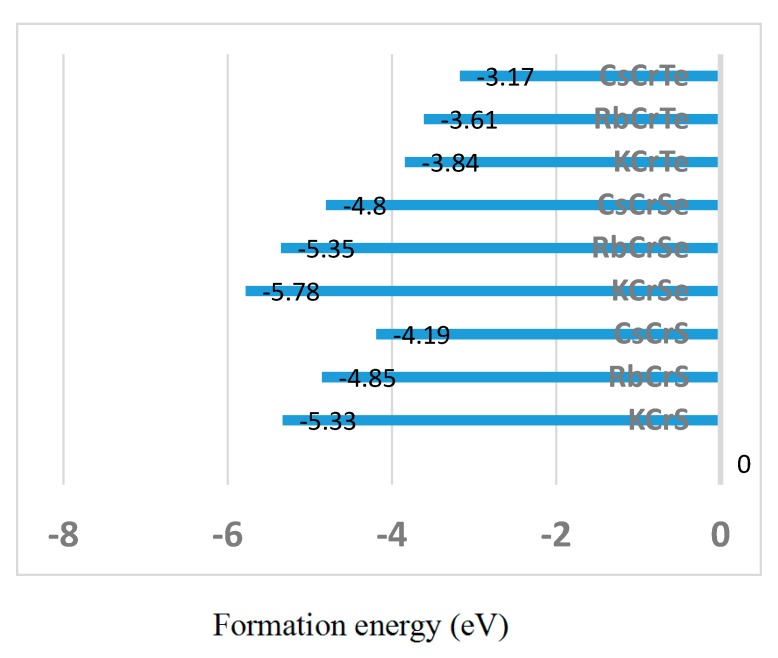
Calculated formation energies (eV) for the XCrZ (X = K, Rb, Cs; Z = S, Se, Te) alloys with the most stable phase.

Our work suggests that the XCrZ (X = Li, K, Rb, Cs; Z = S, Se, Te) half-Heusler alloys are useful in spintonic applications. For the half-Heusler type XCrZ (X = K, Rb, Cs; Z = S, Se, Te) alloys, their formation energies have been calculated based on the following formula:

(2) $$E_{formation}=E_{XCrZ}^{total}-\left(E_{X}^{bulk}+E_{Cr}^{bulk}+E_{Z}^{bulk}\right),$$

where $E_{XCrZ}^{total}$ is the total energy of XCrZ per formula unit, and $E_{X}^{bulk}$, $E_{Cr}^{bulk}$, and $E_{Z}^{bulk}$ are the total energies per atom of each element in the bulk for the X, Cr, and Zr, respectively. The results have been shown in Figure 9; the negative formation energies indicate that these alloys are expected to be stable. Therefore, they have the large change to be synthesized by normal equilibrium methods (e.g., arc-melting). However, for LiCrZ (Z = S, Se, Te), some non-equilibrium methods (e.g., rapid quenching) can be selected to prepare these meta-stable compounds [36].

## 4. Conclusions

A first principles calculation was used to predict a series of new half-Heusler-based, half-metallic materials XCrZ (X = Li, K, Rb, Cs; Z = S, Se, Te) with the largest magnetic moment (5 μ_B_) and large semiconducting-type band-gaps (>2 eV). In detail, for XCrZ (X = K, Rb, Cs; Z = S, Se, Te), the HM nature of these alloys appeared at their equilibrium and strained lattice constants and in their most stable phases. However, for the LiCrZ alloys, the HM behaviors of these alloys did not appear in the most stable phase but in one of the metastable phases. The half-metallicity of the XCrZ alloys is robust against uniform strain, which makes these alloys very stable with respect to the spin polarization properties.

## Figures and Tables

**Table 1:** Optimized lattice constants (a), calculated total and the atomic magnetic moments (M_t_) per formula unit, and sizes of the semiconducting-type band-gap (Gap) at different crystal structures for XCrZ (X = Li, K, Rb, Cs; Z = S, Se, Te). Yes (or no) indicates that this alloy is (or is not) a half metal.

Alloy	Type	a (Å)	M_tot_ (μ_B_)	M_X_	M_Cr_	M_Z_	Gap (eV)	HM Behavior
LiCrS	Type I	5.89	4.82	−0.08	5.04	−0.14	-	No
	Type II	5.82	5.00	−0.08	5.22	−0.12	3.62	Yes
	Type III	5.90	4.97	−0.02	5.18	−0.20	-	No
LiCrSe	Type I	6.19	4.92	−0.08	5.16	−0.16	-	No
	Type II	6.00	5.00	−0.04	5.36	−0.32	3.15	Yes
	Type III	6.11	4.97	0.04	5.30	−0.36	-	No
LiCrTe	Type I	6.60	4.95	−0.02	5.30	−0.32	-	No
	Type II	6.37	5.00	−0.02	5.42	−0.40	2.10	Yes
	Type III	6.60	4.94	0.02	5.40	−0.48	-	No
KCrS	Type I	6.71	5.00	−0.12	5.34	−0.22	3.15	Yes
	Type II	6.90	5.00	−0.32	5.54	−0.24	2.14	Yes
	Type III	6.60	5.00	−0.24	5.40	−0.18	3.41	Yes
KCrSe	Type I	6.91	5.00	−0.26	5.38	−0.12	2.85	Yes
	Type II	6.98	5.00	−0.12	5.58	−0.46	2.09	Yes
	Type III	6.75	5.00	−0.14	5.46	−0.34	3.07	Yes
KCrTe	Type I	7.35	5.00	−0.06	5.46	−0.42	2.87	Yes
	Type II	7.41	5.00	−0.08	5.66	−0.58	1.87	Yes
	Type III	7.11	5.00	−0.10	5.54	−0.46	2.92	Yes
RbCrS	Type I	7.01	5.00	−0.14	5.38	−0.24	2.65	Yes
	Type II	7.21	5.00	−0.30	5.56	−0.26	1.69	Yes
	Type III	6.79	5.00	−0.24	5.40	−0.18	3.19	Yes
RbCrSe	Type I	7.21	5.00	−0.12	5.42	−0.30	2.33	Yes
	Type II	7.49	5.00	−0.08	5.64	−0.56	1.49	Yes
	Type III	6.98	5.00	−0.16	5.46	−0.30	2.72	Yes
RbCrTe	Type I	7.63	5.00	−0.04	5.50	−0.46	2.40	Yes
	Type II	7.81	5.00	−0.06	5.68	−0.62	1.54	Yes
	Type III	7.30	5.00	−0.12	5.54	−0.42	2.71	Yes
CsCrS	Type I	7.35	5.00	−0.14	5.42	−0.28	2.09	Yes
	Type II	7.59	5.00	−0.26	5.58	−0.30	1.41	Yes
	Type III	7.11	5.00	−0.22	5.40	−0.18	2.83	Yes
CsCrSe	Type I	7.54	5.00	−0.02	5.48	−0.46	2.01	Yes
	Type II	7.90	5.00	−0.04	5.68	−0.64	1.31	Yes
	Type III	7.21	5.00	−0.14	5.42	−0.28	2.61	Yes
CsCrTe	Type I	7.98	5.00	−0.02	5.52	−0.50	2.09	Yes
	Type II	8.21	5.00	−0.02	5.72	−0.70	1.31	Yes
	Type III	7.59	5.00	−0.12	5.54	−0.42	2.29	Yes
